# The three‐pillar concept for functional knee positioning in robotic total knee arthroplasty

**DOI:** 10.1002/jeo2.70859

**Published:** 2026-07-20

**Authors:** Alberto Fogacci, Clément Favroul, Cécile Batailler, Elvire Servien, Sébastien Lustig

**Affiliations:** ^1^ Department of Orthopaedics Surgery and Sports Medicine, FIFA Medical Center of Excellence, Croix‐Rousse Hospital Hospices Civils de Lyon Lyon North University Hospital Lyon France; ^2^ Clinica Ortopedica e Traumatologica II IRCCS Istituto Ortopedico Rizzoli Bologna Italy; ^3^ Univ Lyon, Claude Bernard Lyon 1 University, IFSTTAR, LBMC UMR_T9406 Lyon France

**Keywords:** functional knee positioning, knee phenotype, patellofemoral tracking, soft‐tissue balance, total knee arthroplasty

## Abstract

Total knee arthroplasty (TKA) has traditionally relied on standardised alignment targets, yet a relevant proportion of patients continue to report residual symptoms or dissatisfaction despite excellent implant survivorship. Growing recognition of inter‐individual variability in knee phenotype, joint line orientation, ligament behaviour and patellofemoral anatomy has challenged the conventional ‘one‐size‐fits‐all’ paradigm and promoted the development of more personalised strategies. This narrative review summarises the rationale for a more patient‐specific approach to TKA and introduces the concept of functional knee positioning (FKPos) through a practical three‐pillar framework integrating bone morphology, ligament behaviour and patellofemoral biomechanics. FKPos represents an evolution of functional alignment, moving beyond static coronal alignment towards an integrated, three‐dimensional and patient‐specific approach to implant positioning. FKPos is based on three interdependent domains, referred to as the three pillars: bone morphology, ligament behaviour and patellofemoral biomechanics. The bone pillar provides the anatomical foundation by restoring native joint line orientation and three‐dimensional morphology within safe boundaries. The ligament pillar aims to achieve individualised, reproducible gap balance through implant positioning rather than systematic soft‐tissue release. The patellofemoral pillar considers the anterior compartment as a dynamic and measurable component of knee function, with particular attention to trochlear anatomy, anterior offset and intraoperative tracking. CT‐based robotic assistance enables accurate preoperative planning, intraoperative quantification of laxity, controlled adjustment of component position and dynamic assessment of patellofemoral tracking. These technological advances have improved the reproducibility of personalised TKA strategies and allow the three pillars to be assessed within a single operative workflow. Current evidence suggests that FKPos is feasible, safe and associated with satisfactory early clinical outcomes. However, clear superiority over other alignment strategies remains unproven, and long‐term data are still required. The three‐pillar concept provides a practical framework for patient‐specific TKA by integrating bony anatomy, ligament balance and patellofemoral function. Its application should remain within defined safe zones and be interpreted as a functional positioning strategy rather than a universal target.

**Level of Evidence:**

Level V.

## Graphical abstract

The three‐pillar concept for functional knee positioning integrates bone morphology, ligament behaviour and patellofemoral biomechanics into a patient‐specific robotic total knee arthroplasty framework.

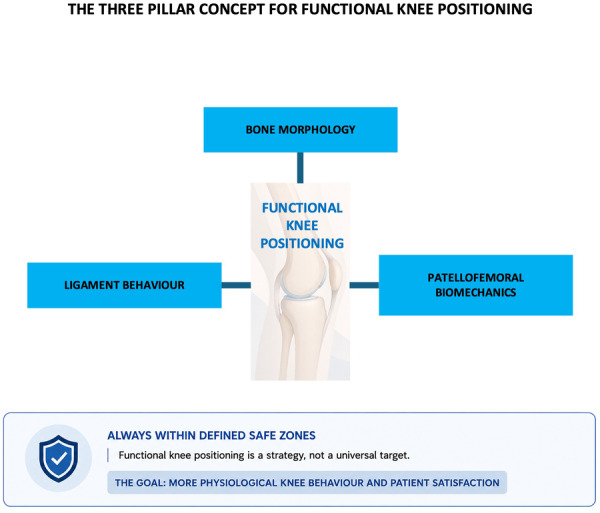

**Abbreviations**
ACL: anterior cruciate ligament
CPAK: coronal plane alignment of the knee
DyAK: dynamic alignment of the knee
FA: functional alignment
FJS: forgotten joint score
FKPos: functional knee positioning
HKA: hip–knee–ankle axis
KA: kinematic alignment
KSS: knee society score
MA: mechanical alignment
mLDFA: mechanical lateral distal femoral angle
mMPTA: mechanical medial proximal tibial angle
OA: osteoarthritis
PCA: posterior condylar axis
PCL: posterior cruciate ligament
rHKA‐90F: robotic hip–knee–ankle angle at 90° of flexion
rKA: restricted kinematic alignment
ROM: range of motion
TEA: transepicondylar axis
TKA: total knee arthroplasty

## BACKGROUND

Total knee arthroplasty (TKA) is a reliable and durable procedure for the treatment of end‐stage knee osteoarthritis (OA), with an estimated implant survival of 82% at 25 years according to a recent systematic review and meta‐analysis by Evans et al. [9].

Despite these favourable long‐term results, dissatisfaction remains clinically relevant, with approximately 10% of patients reporting persistent symptoms or unmet expectations after TKA [5].

This discrepancy between implant survivorship and patient‐perceived outcome has increased interest in factors related to native knee phenotype, soft‐tissue behaviour and functional joint reconstruction.

For decades, mechanical alignment (MA) represented the reference strategy in TKA. Its rationale was to restore a neutral hip–knee–ankle axis (HKA) and a horizontal joint line in order to achieve balanced load distribution and reduce polyethylene wear. Although this approach has demonstrated reproducibility and reliable long‐term outcomes, it remains based on standardised targets and does not fully account for individual anatomical variability.

Increasing evidence has shown wide inter‐individual variability in native limb alignment, joint line orientation and knee morphology. Only a minority of individuals present a truly neutral mechanical axis, while many knees demonstrate constitutional varus or valgus patterns [23].

Consequently, systematic correction towards neutral alignment may result in non‐physiological reconstruction in selected patients, potentially altering knee kinematics, increasing the need for soft‐tissue release, and affecting patient satisfaction.

Classification systems such as the coronal plane alignment of the knee (CPAK) classification were developed to better describe individual coronal phenotypes by integrating constitutional alignment and joint line obliquity [32].

Building upon these principles, there has been a progressive shift towards more personalised alignment strategies, including functional alignment (FA), which aim to integrate three‐dimensional implant positioning with individualised soft tissue balance and preservation of native joint biomechanics across all planes. In this evolving paradigm, a comprehensive approach that simultaneously addresses bone anatomy, ligament balance and patellofemoral mechanics may be required to optimise functional outcomes and patient satisfaction following TKA.

Against this background, the current evidence supporting personalised TKA is synthesised and three pillars concept for functional knee positioning (FKPos) is proposed as a practical conceptual framework integrating bone morphology, ligament behaviour and patellofemoral biomechanics for patient‐specific implant positioning. An illustrative clinical case is included to demonstrate the practical application of three pillars concept within a CT‐based robotic‐assisted workflow.

## FROM A SYSTEMATIC TO A PERSONALISED APPROACH: TOWARDS THE CONCEPT OF FKPos

Historically, TKA philosophy was dominated by systematic alignment strategies, particularly MA, which aimed to restore neutral limb alignment through standardised bone resections. While this approach remains reliable and reproducible, it is inherently based on fixed angular targets that do not consider individual anatomy or the behaviour of the native soft‐tissue envelope [35].

Kinematic alignment (KA) and inverse KA represented early attempts to restore the patient's pre‐arthritic anatomy by resurfacing the joint and preserving native joint line orientation and ligament balance [17, 29]. However, unrestricted restoration of native anatomy may result in excessive alignment outliers, leading to the development of modified approaches such as restricted kinematic alignment (rKA), which aims to individualise component positioning while maintaining predefined safety boundaries [41].

FA has further advanced this concept, particularly with the development of robotic‐assisted surgery. FA combines three‐dimensional implant planning with intraoperative assessment of ligament laxity and flexion–extension gaps, allowing component position to be adjusted in order to obtain a balanced knee while preserving soft tissues [29].

However, the term ‘alignment’ remains conceptually limited. It is often interpreted as a static, two‐dimensional concept focused mainly on the coronal plane. This does not fully reflect knee function, which depends on coronal, sagittal and axial component positioning, as well as ligament behaviour and patellofemoral tracking.

In contrast, the concept of FKPos represents a crucial evolution in TKA philosophy. Rather than aiming to achieve a predefined alignment, FKPos considers implant placement as the result of a dynamic, three‐dimensional process driven by the interaction between bone morphology, ligament tension and joint kinematics. In this framework, implant positioning is not driven by a single predefined angular target. Instead, it is guided by the patient's bony anatomy, the measured laxity envelope and the dynamic behaviour of the patellofemoral joint.

FKPos prioritises the preservation of the native soft tissue envelope, minimising ligament releases, which are often required in traditional alignment techniques, particularly in complex deformities. In this context, the soft tissue envelope can be considered the ‘driving force’ or ‘biological reference’ guiding implant positioning.

**Figure 1:**
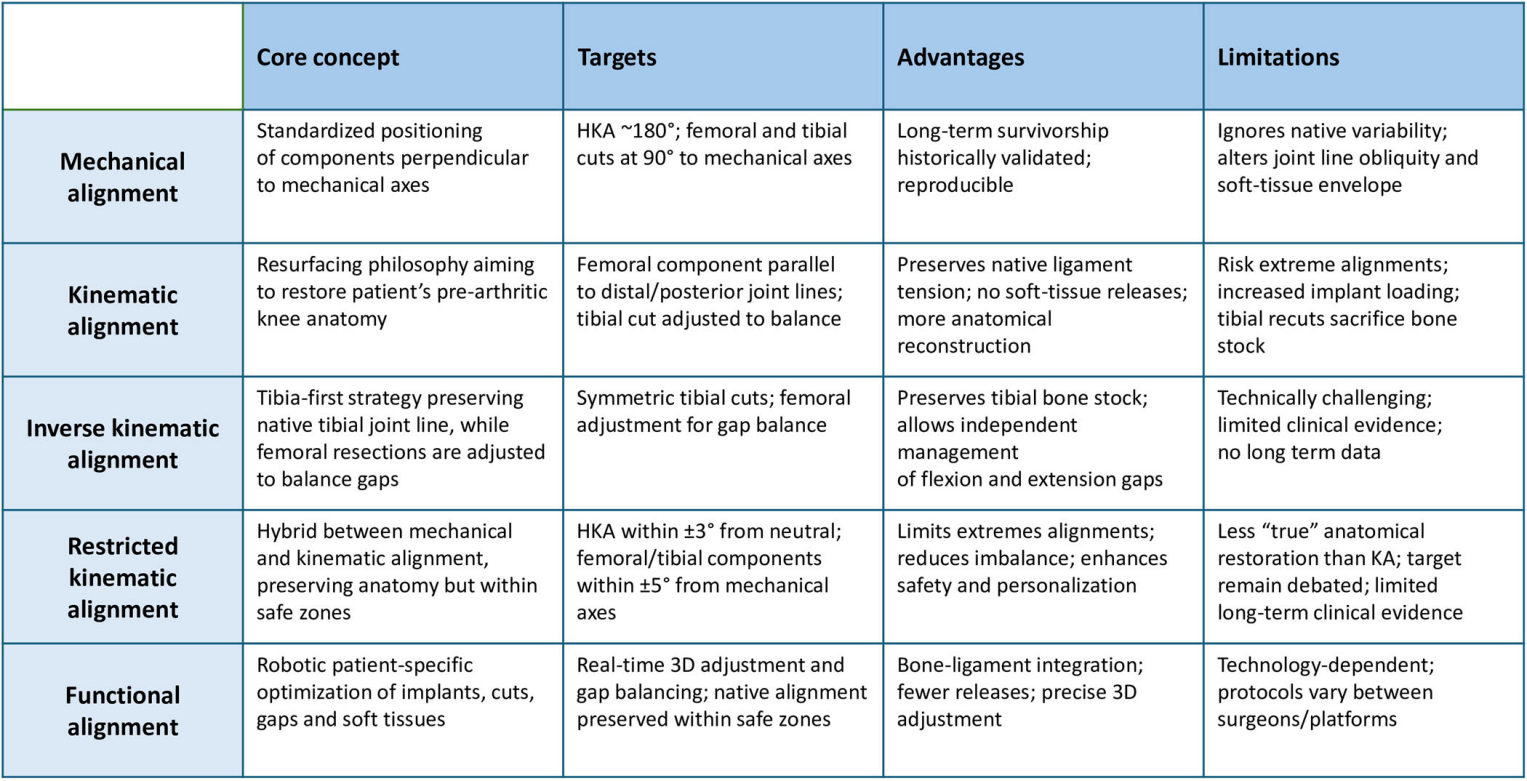
Different type of alignment in total knee arthroplasty.

FKPos therefore represents a transition from static alignment correction towards functional reconstruction of the knee as a complete biomechanical system [25] (Figure 1).

## RATIONALE FOR THE THREE‐PILLAR CONCEPT

The native knee is characterised by substantial anatomical and functional variability. This variability affects not only the coronal plane but also sagittal and axial morphology, ligament laxity and anterior compartment anatomy. The three‐pillar concept was developed to organise these sources of variability into a practical framework for robotic‐assisted TKA.

### Bone morphology

Three‐dimensional analyses of non‐osteoarthritic knees have demonstrated wide variation in femoral and tibial joint line orientation and multiple combinations of joint line obliquity [16].

In osteoarthritic knees, alignment variability remains substantial, with patterns that differ from young non‐arthritic knees, including more frequent varus femoral alignment and relatively neutral tibial orientation [38]. These findings support the absence of a single physiological alignment target.

This variability is not limited to the coronal plane. Sagittal parameters such as tibial slope, distal femoral flexion and combined sagittal alignment also influence knee kinematics and gap behaviour. Similarly, rotational morphology varies considerably between individuals. Hess et al. demonstrated that similar coronal phenotypes may present different rotational phenotypes, with complete congruence of the distal femoral, posterior femoral, anterior trochlear and proximal tibial joint lines observed in only 2.3% of non‐osteoarthritic knees [13]. These data confirm that knee morphology is intrinsically multiplanar.

Robotic data have also shown that coronal alignment changes during flexion in most knees, with only approximately 14% maintaining a consistent pattern throughout ROM. The robotic HKA angle at 90° of flexion (rHKA‐90F) allows assessment of alignment in flexion and integrates the effects of femoral rotation, tibial orientation and soft‐tissue behaviour [1].

The dynamic alignment of the knee (DyAK) classification further expands this concept by integrating alignment in extension and flexion into a dynamic framework. This approach emphasises that knee alignment evolves throughout the ROM and should be interpreted as a functional, patient‐specific characteristic rather than a fixed coronal parameter [2].

These findings emphasise that alignment should not be considered a fixed static parameter, but a functional and patient‐specific pattern expressed throughout ROM.

### Ligament behaviour

Ligament behaviour is a major source of variability in knee function. Unlike bone morphology, which can be accurately characterised using imaging, the soft‐tissue envelope is dynamic and changes throughout ROM. Quantitative analyses using computer‐assisted surgery have shown that ligament tension patterns differ substantially between patients, even within the same deformity group.

In varus osteoarthritic knees, a ‘standard’ ligament pattern, defined by greater lateral laxity in both extension and flexion and a larger flexion than extension gap, was observed in only approximately 65% of cases [12]. Furthermore, although the degree of varus deformity correlates with mediolateral gap difference in extension, this relationship disappears in flexion, where ligament behaviour becomes less predictable [12].

More recently, the concept of laxity phenotypes has emphasised that each knee presents a specific combination of medial and lateral laxity in extension and flexion, only weakly correlated with overall limb alignment [11].

These observations indicate that static alignment and bony morphology alone cannot reliably predict soft‐tissue behaviour. Therefore, individualised intraoperative assessment of ligament laxity is essential when aiming to restore physiological balance without systematic soft‐tissue release.

### Patellofemoral anatomy and tracking

The anterior compartment represents a distinct source of anatomical and functional variability. Anatomical studies have shown that commonly used femoral reference axes, including the surgical epicondylar axis, do not consistently correspond to distal femoral geometry [28].

Furthermore, anterior morphology is closely related to posterior condylar anatomy. In conditions such as trochlear dysplasia, alterations of the trochlea are associated with significant changes in posterior condylar geometry, including an increased posterior condylar angle due to relative shortening of the lateral condyle [37]. These data indicate that anterior and posterior compartments are anatomically interdependent and cannot be considered independently.

From a functional perspective, even small deviations from native trochlear anatomy may result in patellar maltracking, anterior knee pain and suboptimal outcomes. Recent studies have demonstrated that restoration of the anterior compartment remains challenging, and that variations in trochlear offset and patellar thickness across the flexion arc may significantly influence postoperative function [21].

The patellofemoral joint should therefore be considered as a third functional space of the knee. Its optimisation requires assessment of trochlear morphology, anterior offset, patellar thickness and dynamic tracking, rather than relying exclusively on tibiofemoral alignment or gap balance.

## THE THREE PILLARS OF FKPos

FKPos is based on the principle that successful TKA requires integration of three interdependent domains: bone morphology, ligament behaviour and patellofemoral biomechanics. These domains cannot be treated independently. A modification in coronal or rotational component positioning may affect tibiofemoral balance and patellofemoral tracking. Similarly, attempts to optimise ligament balance may influence joint line orientation or anterior compartment mechanics.

**Figure 2:**
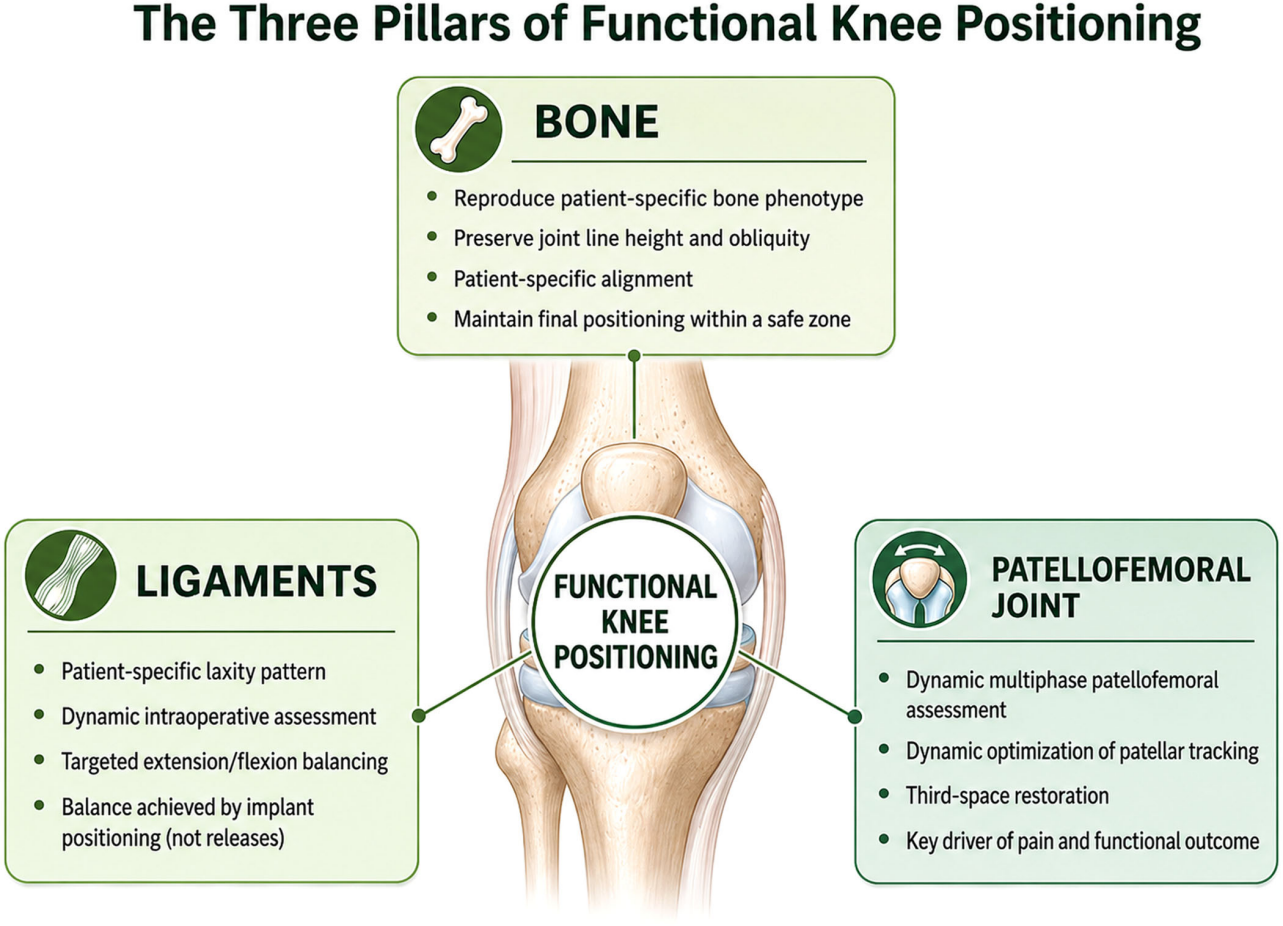
Conceptual framework of functional knee positioning (FKpos) based on the three pillars: bone, ligament and patellofemoral joint.

The three‐pillar concept provides a practical framework for translating personalised alignment into functional implant positioning. Image‐based robotic technology facilitates this process through three‐dimensional planning, intraoperative measurement of laxity, predictive gap analysis and dynamic patellofemoral assessment (Figure 2).

### Bone

Within the three pillars framework, the bone component represents the primary structural determinant of FKPos, as it forms the foundation for ligament balance and patellofemoral mechanics. The objective is to restore patient‐specific bone anatomy within defined safety boundaries, integrating coronal, sagittal and axial planes.

In the coronal plane, this means reproducing the native phenotype rather than imposing neutral alignment. Femoral and tibial resections follow mLDFA and mMPTA, maintaining joint line obliquity, which is essential for more physiological load distribution [40]. Joint line height should also be preserved, as excessive elevation, particularly proximalization, has been associated with mid‐flexion laxity [31, 40].

In the sagittal plane, bone positioning aims to restore the native relationship between femoral flexion and tibial slope, given their combined influence on flexion gap behaviour [43]. The femoral component is positioned to respect distal femoral curvature and avoid anterior notching, typically within a controlled degree of flexion. Tibial slope is reproduced according to the patient's native anatomy but remains constrained by implant design. Careful control of this combined sagittal alignment is essential to avoid excessive flexion positioning of the components.

In the axial plane, rotational alignment is treated as a functional variable rather than a fixed anatomical reference. Although traditional landmarks such as the transepicondylar axis (TEA), posterior condylar axis (PCA) and tibial anteroposterior axis provide a baseline, final rotational positioning is adapted within safe boundaries to accommodate individual variability and to optimise flexion space gap, recognising that rigid application of standard references may lead to flexion imbalance [40].

A key principle of this first pillar is that bone resection characteristics are the primary determinants of tibiofemoral gap behaviour and balance. In FKPos, tibiofemoral gaps are predicted and adjusted through implant positioning and bone resections, reversing the traditional ligament‐driven approach based on the soft tissue release. This strategy is enabled by three‐dimensional planning and intraoperative assessment, allowing accurate control of gap changing.

Implant sizing supports this concept following native femoral anatomy restoration, avoiding overhang and patellofemoral overstuffing, thereby linking bone reconstruction to anterior compartment function.

All adjustments are performed within defined safety boundaries (coronal, sagittal, rotational alignment and joint line) to respect anatomical variability without exceeding mechanical limits.

The bone pillar represents a personalisation process, providing a stable and reproducible foundation for ligament balance and patellofemoral tracking.

### Ligament

In FKPos, ligament balance is a measurable and flexion‐dependent parameter, reflecting the patient‐specific soft‐tissue envelope. Native knees, particularly in varus morphotypes, exhibit substantial variability in laxity with physiological asymmetry between compartments and increased laxity in flexion, requiring an individualised treatment [12].

Soft‐tissue balancing is a central principle in FKPos. A balanced knee is defined by a mediolateral gap difference ≤1–1.5 mm, with the final gaps in full extension and at 90° of flexion not exceeding ±2 mm from the target implant thickness. A slight lateral laxity in flexion (≈1 mm greater than medial compartment) is considered physiological. These thresholds are critical, as deviations beyond this range—particularly excessive medial laxity—have been associated with inferior clinical outcomes and increased pain [40].

A key technical feature of FKPos is that ligament balance is achieved primarily before bone cuts, through predictive gap analysis and implant position refinement, rather than through secondary soft‐tissue release. Robotic studies have shown that bone‐based resurfacing alone does not reliably achieve ligament balance. In varus knees, KA achieved target gaps in only 65.7% of extension and 49.1% of flexion cases, whereas incorporating soft‐tissue laxity into implant positioning according to functional principles enabled more consistent gap balancing with minimal soft‐tissue release [39].

However, balanced gaps in extension and at 90° of flexion do not guarantee balanced behaviour throughout the entire ROM. Even when using functional positioning, a substantial proportion of knees may remain unbalanced in mid‐flexion, highlighting the non‐isometric behaviour of ligaments and the influence of joint line position, femoral geometry and implant design [8].

Therefore, FKPos should not be reduced to two‐point gap balancing, but should aim to reconstruct a stable and functional laxity envelope throughout the flexion arc.

Cruciate management and implant design also influence ligament balance. PCL resection increases the flexion gap, particularly laterally and may affect mediolateral balance, requiring compensatory adjustments in component position [24].

The ligament pillar in FKPos aims to reconstruct the patient‐specific laxity envelope through coordinated implant positioning. The objective is to achieve stable extension, physiological flexion laxity and minimal mediolateral flexion imbalance, ensuring a reproducible mechanical environment for optimal knee function.

### Patellofemoral joint

The patellofemoral joint represents the third pillar of FKPos and is a critical yet historically underestimated determinant of postoperative outcomes.

The anterior compartment can be directly assessed and dynamically optimised intraoperatively, particularly with image‐based robotic assistance.

The patellofemoral biomechanics is highly sensitive to implant positioning, trochlear geometry and anterior offset restoration, and even small deviations can result in maltracking, anterior knee pain and functional impairment [30].

Restoration of the anterior femoral offset and trochlear anatomy is the key, which directly influences patellar tracking and extensor mechanism efficiency.

Robotic systems allow precise positioning of the prosthetic trochlea relative to the native anatomy using CT‐based planning, enabling accurate reproduction of trochlear depth, orientation and curvature. This is critical because conventional instrumentation lacks the ability to reliably restore the anterior compartment [10].

Quantitative assessment of trochlear offset can be performed at multiple flexion angles (0°, 30°, 70°, 90°), demonstrating that both under‐ and overstuffing occur throughout the ROM, with overstuffing—especially in mid‐ to deep flexion—being associated with reduced flexion and inferior functional outcomes [21].

In this context, global anterior compartment restoration, defined as the combined effect of trochlear offset and patellar thickness, becomes essential. Overstuffing increases patellofemoral contact pressures, tightens the extensor mechanism and limits flexion, whereas excessive understuffing may reduce quadriceps efficiency and alter tracking [21].

Safe ranges have been proposed for key parameters such as patellar tilt (0°–5° medial opening), patellar translation (±2 mm) and patellar offset (ΔPO between 0 and −5 mm), aiming to avoid both overconstraint and instability of the patellofemoral articulation [6]. While restoration within these safe zones does not necessarily translate into superior short‐term clinical outcomes, it significantly influences intraoperative decision‐making, particularly regarding the need for patellar resurfacing [6].

Robotic‐assisted surgery also allows dynamic intraoperative assessment of patellar tracking. Using a tracked reference point on the patella, the system can record patellar position throughout ROM, allowing three‐dimensional reconstruction of the tracking path and comparison between native, trial and final implant conditions [3]. This enables real‐time evaluation of mediolateral translation and anteroposterior position, with accuracy within 1 mm, and allows identification of subtle tracking abnormalities that would otherwise remain undetected [3].

This dynamic assessment transforms the anterior compartment into a measurable and feedback‐driven domain. Component rotation, mediolateral positioning, patellar resection thickness and patellar button position can be adjusted when tracking is suboptimal. Sequential assessment before bone cuts, after trial implantation and after final component implantation may therefore help optimise the third space [36].

The patellofemoral domain represents a dynamically assessable component in TKA, integrating restoration of anterior offset, accurate trochlear reconstruction, and real‐time intraoperative patellar tracking assessment. This approach enables patient‐specific optimisation of the extensor mechanism and patellofemoral kinematics, addressing a key source of postoperative pain and suboptimal clinical outcomes.

## FROM ALIGNMENT TARGETS TO POSTOPERATIVE KNEE MOTION

Although FKPos incorporates alignment targets, bone resections, gap assessment and predefined safe boundaries into the planning process, these variables should be viewed as surrogate intraoperative parameters intended to optimise postoperative knee function rather than as primary surgical objectives. The primary aim of FKPos is not the reproduction of predefined angular targets, but the restoration of a knee that behaves as physiologically as possible throughout the range of motion. Native knee kinematics are characterised by a relatively stable medial compartment that functions as a pivot point while most anteroposterior translation occurs in the lateral compartment [14, 19]. During flexion, the lateral femoral condyle progressively translates posteriorly through a combination of rolling and sliding mechanisms, generating femoral rollback and contributing to greater flexion efficiency and physiological load distribution [33]. This asymmetric compartmental motion is coupled with axial rotation, with posterior translation of the lateral femoral condyle producing progressive internal rotation of the tibia relative to the femur during flexion [20]. These kinematic patterns continuously evolve throughout the range of motion as a consequence of the interaction between articular morphology and soft‐tissue constraints rather than being determined by a single static alignment configuration [27, 33].

Within the FKPos framework, bone morphology determines joint line orientation and the three‐dimensional anatomical relationships that influence compartmental motion; ligament behaviour modulates femoral rollback and axial rotation by defining the functional laxity envelope; and patellofemoral biomechanics help ensure that tibiofemoral adjustments remain compatible with physiological patellar tracking and extensor mechanism function. Within this perspective, component positioning, gap management and soft‐tissue balancing should be interpreted as means of creating the anatomical and biomechanical conditions that favour more physiological postoperative knee motion rather than as objectives in themselves.

## SURGICAL WORKFLOW: APPLICATION OF THE THREE‐PILLAR CONCEPT

The application of the three‐pillar concept within a CT‐based robotic‐assisted TKA (MAKO system, Stryker®) workflow is illustrated through the following clinical case.

A 75‐year‐old male patient (body mass index of 24.5 kg/m^2^) presented with a 6‐year history of progressive left knee pain, reduced walking tolerance and difficulty descending stairs, ultimately leading to significant functional limitation.

Clinical examination showed a dry knee, ROM from 0° to 120°, no antero‐posterior and coronal instability and diffuse joint line tenderness.

**Figure 3:**
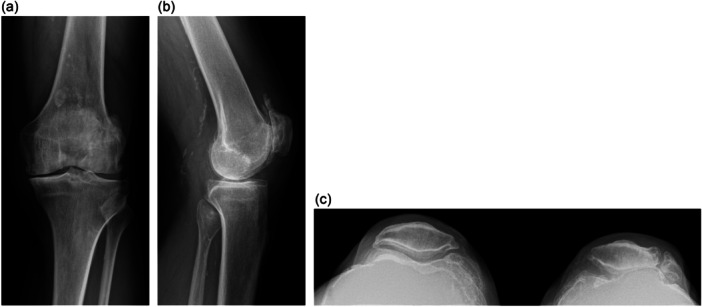
Clinical case: preoperative radiographs. (a) Weight‐bearing anteroposterior radiograph. (b) Lateral radiograph. (c) Axial patellofemoral (Merchant) views.

Preoperative radiographs demonstrated bicompartmental femorotibial osteoarthritis predominantly involving the medial compartment (Kellgren–Lawrence grade III), associated with severe patellofemoral involvement (Iwano grade IV). Lower‐limb alignment analysis showed a preoperative HKA angle of 173°, with an mLDFA of 89.0°, mMPTA of 85.0°, and tibial slope of 7°. Patellofemoral assessment demonstrated lateral patellar tilt (13.5°), lateral patellar displacement (6.5 mm) and a positive congruence angle (+23°), consistent with lateral patellar maltracking. The Caton–Deschamps index was 0.89 (Figure 3).

**Step 1: CT‐based planning and bone phenotype assessment: Bone pillar**

Preoperative CT‐based planning aimed to define patient‐specific implant positioning according to the native bony phenotype through an integrated multiplanar assessment.

On the femoral side, sagittal planning defined component size and flexion to avoid anterior notching and overstuffing while maintaining congruence with the anterior cortex and posterior condyles. Axial planning adjusted component rotation according to anatomical landmarks with particular attention to reproducing native trochlear anatomy, whereas coronal alignment and distal resection were tailored to the native morphotype to preserve joint‐line orientation and height.

On the tibial side, coronal alignment was adapted to native anatomy, axial rotation and sizing were planned using the Akagi line while avoiding overhang, and posterior slope was defined within the limits of implant design.

Component sizing resulted from the integrated assessment of all planes.

The key preoperative parameters, initial implant planning and planned bone resections are summarised in Table 1.

**Table 1:** Key preoperative parameters, initial implant planning and planned bone resections.

Category	Parameter	Value
Preoperative alignment and morphology	HKA	173°
	mLDFA	89.0°
	mMPTA	85.0°
	Tibial slope	7°
Initial planned alignment	Planned HKA	0°
Femoral component planning	Coronal alignment	2° valgus
	Axial rotation	0.5° external rotation relative to the TEA
	Sagittal alignment	5.6° flexion
Tibial component planning	Coronal alignment	2° varus
	Sagittal alignment	3° posterior slope
Implant selection	Implant	Triathlon CS, PCL‐retaining
	Femoral component size	Size 5
	Tibial component size	Size 5
	Insert thickness	9 mm
Initial planned bone resections	Distal femur	6.0 mm lateral/7.5 mm medial
	Posterior femur	7.0 mm lateral/8.0 mm medial
	Proximal tibia	6.0 mm lateral/6.0 mm medial

Abbreviations: HKA, hip–knee–ankle angle; mLDFA, mechanical lateral distal femoral angle; mMPTA, mechanical medial proximal tibial angle; PCL, posterior cruciate ligament; TEA, transepicondylar axis.

**Figure 4:**
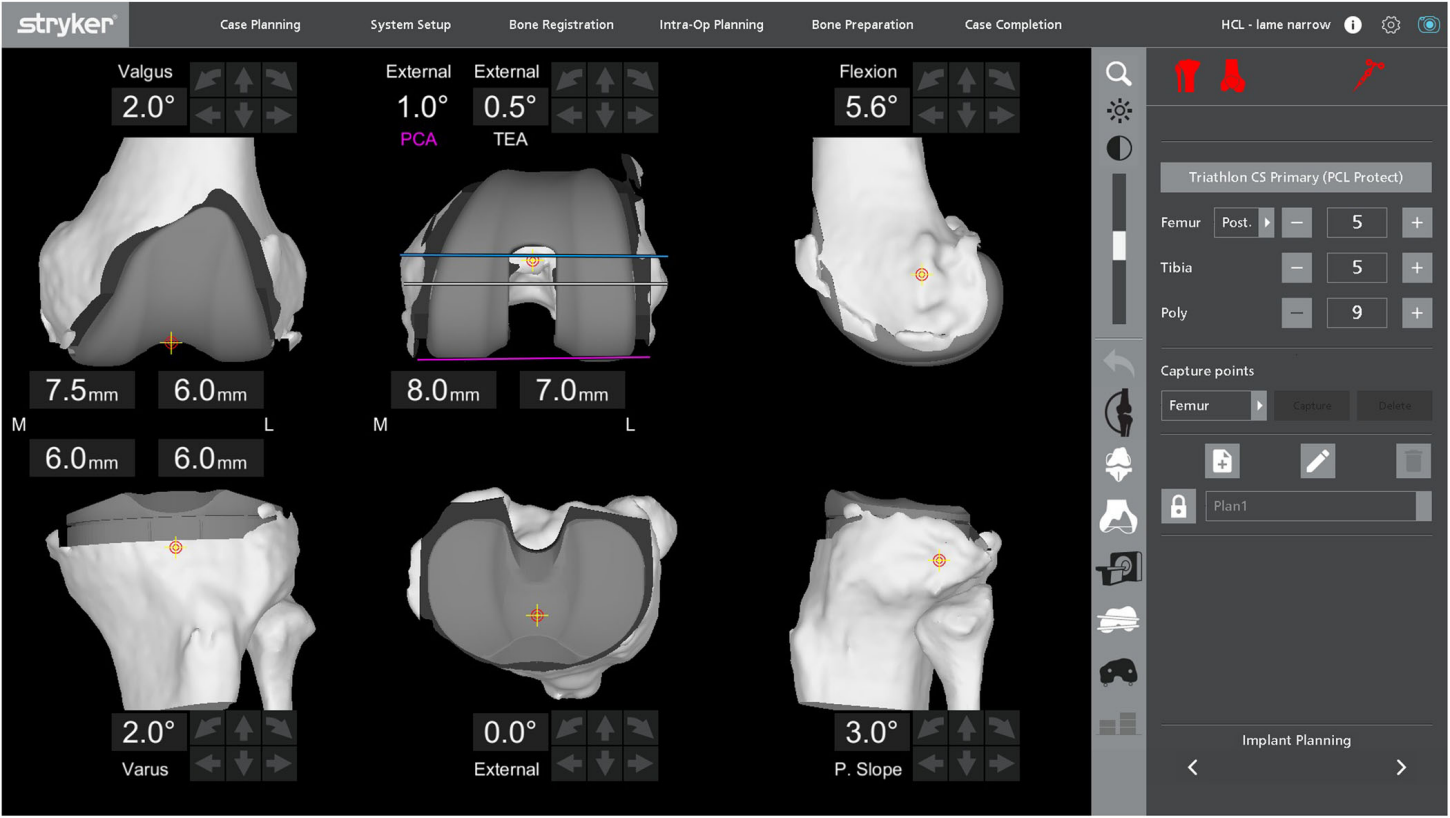
Bone pillar. Preoperative CT‐based planning of the reported case, showing implant positioning and planned bone resections.

These planned resections reflected the need to compensate for asymmetric wear while maintaining a patient‐specific joint line. However, this bone‐based plan represented only the starting point and was subsequently challenged intraoperatively by objective laxity assessment (Figure 4).

**Step 2: Native anatomy and laxity assessment**

After bone registration, native knee kinematics were dynamically assessed to quantify deformity and soft‐tissue behaviour throughout the ROM. Native alignment and its correctability were first evaluated under manual stress to determine the degree of deformity reducibility and thereby guide the relative contribution of bone resection and soft‐tissue balancing. Intraoperative laxity was then recorded in extension and flexion, providing a quantitative assessment of medial and lateral compartment behaviour and allowing refinement of the implant plan to achieve balanced gaps while preserving the native soft‐tissue envelope.

**Figure 5:**
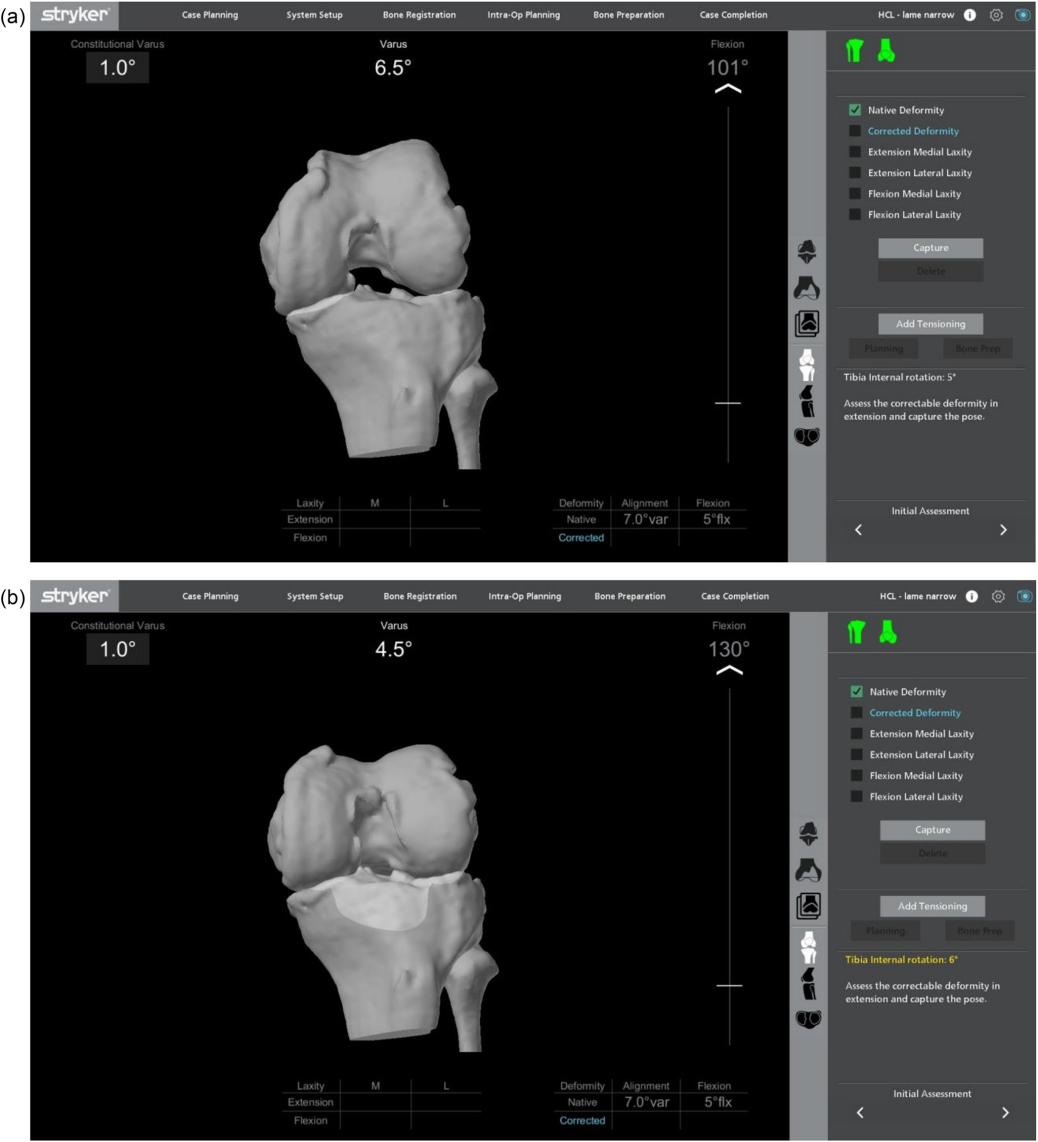
Dynamic change of coronal alignment during knee flexion. (a) Coronal alignment at 101° of flexion showing 6.5° varus. (b) Coronal alignment at 130° of flexion showing 4.5° varus, showing reduction of varus with increasing flexion.

In this case, the knee showed 7° of varus alignment in full extension. With increasing flexion, alignment remained in varus but showed a progressive reduction, measuring 6.5° at 101° of flexion and 4.5° in deep flexion. This pattern highlights a clear discrepancy between extension and flexion alignment. Coronal alignment is dynamic rather than static and evolves throughout the ROM [1] (Figure 5).

**Step 3: Gap balancing and implant position refinement: Ligament pillar**

Initial laxity assessment showed a medial gap of +0.5 mm and lateral gap of +3.5 mm in extension. At 90° of flexion, the medial gap was −1.5 mm and the lateral gap +3.0 mm. The objective of intraoperative balancing was to obtain a tight and symmetric extension gap (0 mm) and a slightly increased flexion gap with controlled mediolateral asymmetry (0.5 and 1.5 mm).

The selected extension‐flexion targets were also influenced by the condylar‐stabilising implant adopted, with preservation of the PCL. Unlike posterior‐stabilised designs, in which tighter flexion targets are pursued after PCL resection, retention of the PCL allows acceptance of a slightly increased flexion gap and controlled lateral laxity to follow a more physiological soft‐tissue tensioning target.

Starting from extension balancing, tibial varus was increased by 2° in order to reduce the lateral extension gap, while femoral valgus was decreased by 1.5° for the same purpose.

In flexion, medial gap opening was achieved through the combined effect of slight anteriorization of the femoral component (approximately 1 mm through increased posterior condylar resection), carefully evaluated relative to the native trochlear anatomy in order to avoid anterior compartment overstuffing, and additional external femoral rotation (0.5° relative to the TEA). This rotational adjustment also contributed to improvement of patellofemoral maltracking. The previous tibial adjustment further contributed to partial closure of the lateral flexion gap and overall mediolateral balancing.

Final femoral flexion and posterior tibial slope remained unchanged from the preoperative plan, with no evidence of anterior femoral notching.

**Figure 6:**
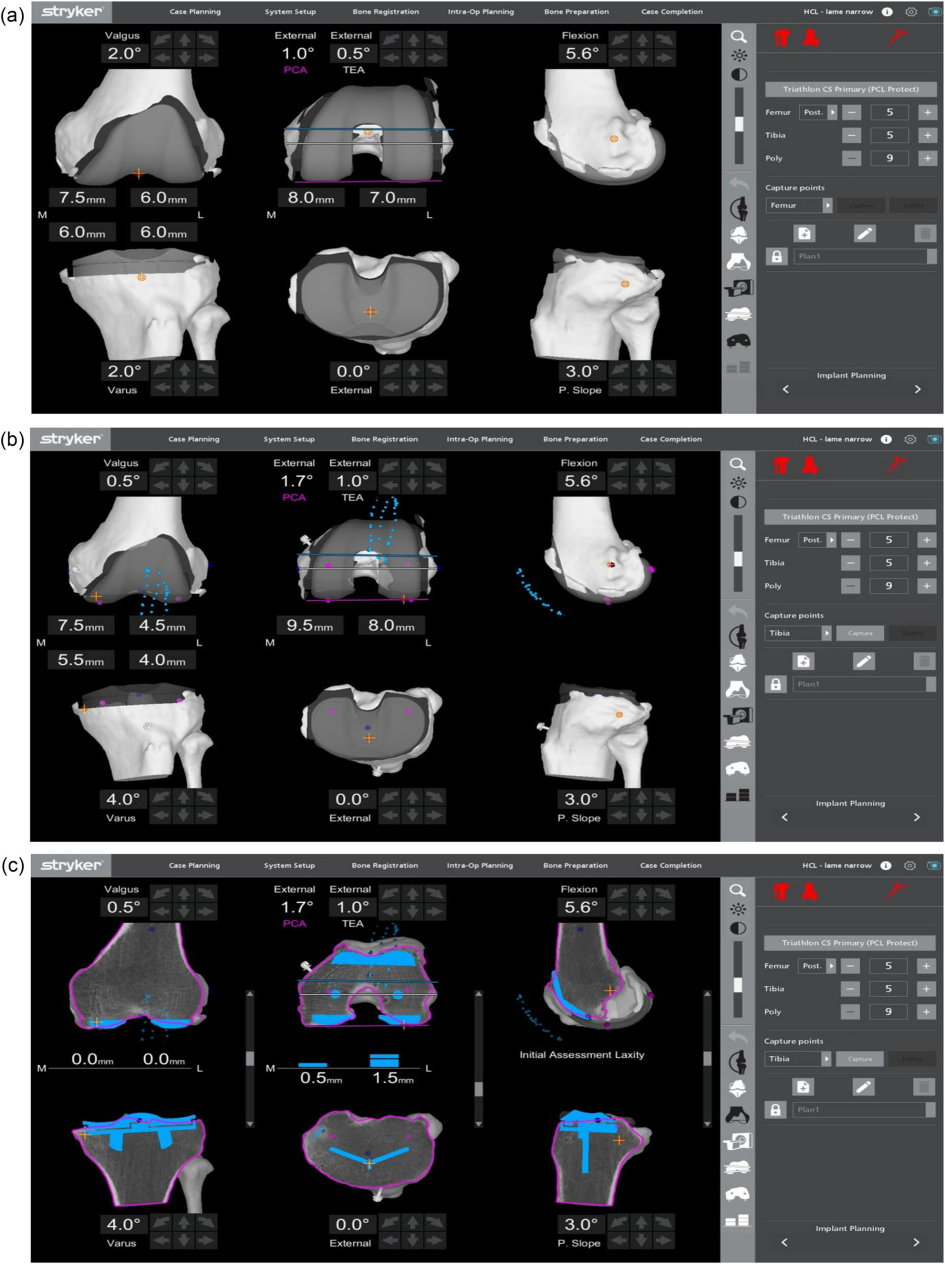
Ligament pillar. Intraoperative gap assessment and final implant positioning. (a) Preoperative CT‐based planning showing initial implant positioning and predicted bone resections. (b) Final implant positioning after adjustment according to extension and flexion gap assessment. (c) Final gap configuration demonstrating a tight and symmetrical extension space and controlled mediolateral asymmetry in flexion.

Collectively, these modifications restored balanced gaps through component positioning alone, without the need for soft‐tissue release (Figure 6).

**Step 4: Patellofemoral pillar**

The patellofemoral joint assessment represents a key step in the functional positioning workflow, ensuring that the reconstructed third space allows physiological tracking.

Patellar tracking is recorded by dynamic assessment from full extension to 90° of flexion. The evaluation is performed first on the native joint to establish a baseline, then after bone resections with trial components in place, and finally—when indicated—after patellar resurfacing with the trial component in situ.

This sequential evaluation allows the surgeon to integrate the effects of bone resections and component positioning on patellofemoral biomechanics, enabling physiological restoration of the third space.

In the present case, the initial assessment (native knee anatomy) demonstrated a lateralized patellar tracking pattern during early and mid‐flexion. Based on this finding, femoral component positioning was refined during the planning phase, including a slight increase in external rotation and lateralisation to improve patellar engagement within the trochlear groove.

After implant planning and bone resections, a second assessment was performed with trial components in place and prior to patellar resurfacing. This intermediate evaluation showed a clear improvement in patellar tracking, with partial medialization and better engagement within the trochlear groove.

A final assessment was then performed after patellar resurfacing using the same landmark and methodology. Medialization of the patellar button was performed to further optimise patellofemoral alignment, resulting in a centralised and physiological patellar pathway throughout the range of motion, with additional reduction of lateral translation.

In the coronal plane, these changes reflected progressive medialization of the patella and restoration of patellofemoral congruence. In the sagittal plane, the anterior patellofemoral offset was preserved across all three assessments, as confirmed by the superimposable tracking curves. Overall, the correction of patellar tracking was achieved through a stepwise optimisation of femoral component positioning followed by patellar resurfacing, without the need for additional soft‐tissue procedures.

**Figure 7:**
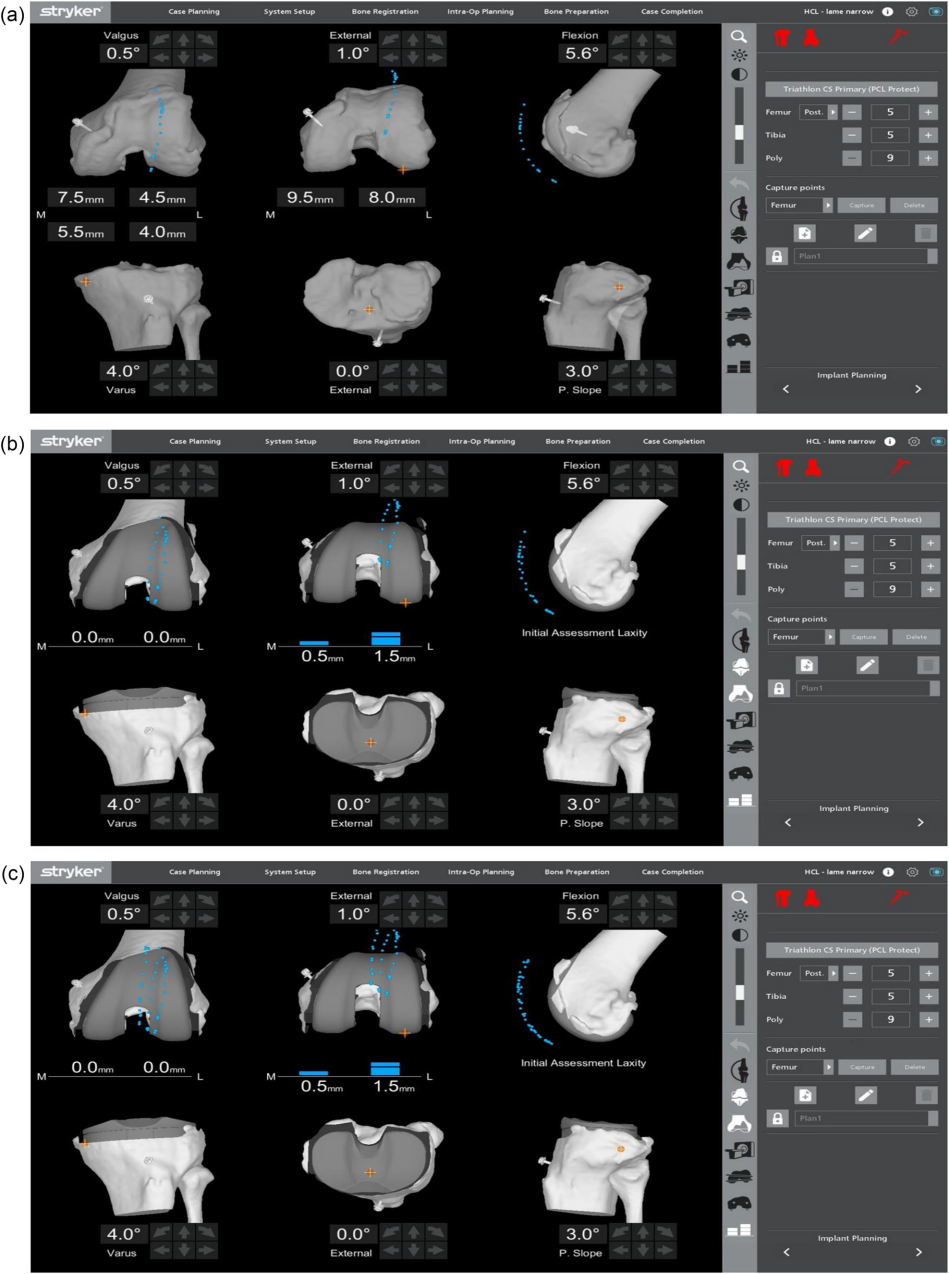
Patellofemoral pillar. Sequential assessment of patellar tracking. (a) Native patellofemoral kinematics. A lateralized patellar tracking pattern is observed during early and mid‐flexion, with reduced engagement within the trochlear groove. (b) Assessment after bone resections and trial implantation showing improved trochlear engagement and partial medialization following refinement of femoral component positioning. (c) Final assessment after patellar resurfacing demonstrating a centralised and physiological patellar pathway with restoration of patellofemoral congruence. Preservation of the anterior patellofemoral offset is confirmed by the superimposable tracking curves.

From a decision‐making perspective, intraoperative findings guide patellar management: when tracking is suboptimal, component positioning should be refined, and, if necessary, resurfacing may be considered, with attention to patellar resection thickness and button positioning. This decision also depends on the intraoperative status of the patellar cartilage and the preoperative clinical presentation, particularly anterior knee pain (Figure 7).

**Step 5: Final validation and implant implantation**

Trial components confirmed global knee stability, balanced gaps and satisfactory patellofemoral tracking throughout the range of motion. Final components were therefore implanted according to the validated plan without further adjustments.

Final extension alignment assessment was 4° of varus while the flexion alignment at 90° revealed an rHKA‐90F of 5.5° of varus.

Compared with the preoperative rHKA‐90F of 6.5° varus, this reflected partial correction towards neutrality while preserving moderate residual varus in flexion. This value lies within the range previously associated with improved functional outcomes and may reflect preservation of physiological soft‐tissue tension and a more native flexion kinematic pattern. [13]

These findings illustrate the combined effect of bone resection and soft‐tissue balancing and further emphasise that alignment in flexion may differ from extension and should be specifically considered during intraoperative decision‐making. Rather than enforcing a fixed neutral target throughout the range of motion, FKPos aims to achieve a stable knee with kinematic characteristics that more closely approximate physiological function [13].

## FEASIBILITY, BOUNDARIES AND CONTROVERSIES OF FKPos

FKPos is a personalised approach aimed at integrating bone morphology, ligament balance and patellofemoral mechanics. However, its application must remain within defined safety boundaries. Large population‐based analyses have shown substantial variability in native knee alignment, supporting individualised implant positioning rather than systematic neutral alignment. Nevertheless, extreme phenotypes should not be reproduced without restriction [15].

The safe‐zone concept is therefore central to FKPos. It represents a multidimensional range that includes coronal alignment, sagittal alignment, axial rotation, joint line orientation, joint line height and soft‐tissue balance, ensuring a balance between anatomical restoration and implant longevity [7].

Moderate deviations from traditional alignment targets, such as tibial component varus greater than 3°, may be safe when integrated into a balanced functional reconstruction, but isolated angular thresholds should not be interpreted independently of the global construct [26].

Several controversies remain. First, restoring patient‐specific anatomy may be inappropriate when the preoperative anatomy reflects pathological adaptation rather than constitutional morphology. Second, ligament targets are not universal and depend on implant design, constraint, cruciate management and surgeon preference. Third, optimisation of one pillar may compromise another. For example, component rotation selected to improve flexion balance may affect patellofemoral tracking, and restoration of coronal anatomy may produce unacceptable ligament asymmetry.

Another important consideration is that the ability of FKPos to restore physiological knee kinematics is also influenced by implant design. The rationale of FKPos is to shift from universal alignment targets towards a patient‐specific strategy according to bone morphology, soft‐tissue behaviour and patellofemoral biomechanics. The prosthetic design should also be regarded as part of the personalisation process, since different patients may benefit from different implant kinematic philosophies. However, prosthesis selection often remains dictated by surgeon preference, institutional practice, or the implant options available within a given robotic system, potentially limiting the ability to fully individualise reconstruction.

From a biomechanical perspective, implant geometry substantially influences postoperative knee kinematics by modulating anteroposterior stability, femoral rollback, tibiofemoral axial rotation and load transmission [34]. Cruciate‐retaining designs depend on a competent PCL to contribute to flexion stability and femoral rollback, whereas posterior‐stabilised implants substitute this function through a cam‐post mechanism. Ultracongruent or condylar‐stabilising inserts increase intrinsic stability through greater conformity [34], while medial‐stabilised or medial‐pivot designs seek to reproduce native asymmetric knee kinematics by combining relative medial stability with greater lateral compartment mobility [18].

Consequently, identical alignment targets, gap characteristics and patellofemoral optimisation may not necessarily translate into identical postoperative kinematics across different prosthetic designs. Implant geometry not only influences the kinematic behaviour of the reconstructed knee but may also constrain the reconstructive strategies that can reasonably be pursued, as alignment targets, gap management and soft‐tissue balancing strategies may vary according to the prosthetic philosophy being used [18, 34]. In this sense, the three pillars define the anatomical and functional objectives of reconstruction, whereas implant design influences both how these objectives are implemented and how they are ultimately expressed after implantation. Accordingly, a perfectly positioned implant may still fail to reproduce physiological knee motion if the selected prosthetic design does not permit the intended kinematic behaviour [18]. Therefore, the three‐pillar framework should not be regarded as independent concept from the implant design chosen. Instead, its application should be interpreted in the context of the biomechanical characteristics and kinematic philosophy of the selected prosthesis.

FKPos is also highly dependent on technology. CT‐based robotic systems provide precise planning and intraoperative measurements, but accuracy depends on image segmentation, registration, stress testing and interpretation of robotic data. Moreover, most available clinical studies report short‐term outcomes and long‐term data on implant survivorship, polyethylene wear, instability and revision risk remain limited.

Therefore, FKPos should be considered a structured and reproducible framework for personalised TKA, not a universal solution.

## CLINICAL EVIDENCE

Current evidence suggests that FKPos is feasible, reproducible and associated with satisfactory short‐term clinical outcomes. However, clear superiority over other alignment strategies has not been consistently demonstrated.

Recent data have shown good functional outcomes and high satisfaction after robotic‐assisted TKA performed within a functional safe zone, even when native CPAK phenotype is modified [4]. These findings suggest that achieving a stable and balanced reconstruction may be more important than strictly reproducing preoperative coronal alignment.

Comparative studies have also suggested potential benefits of FKPos on patient perception of the joint. Kafelov et al. reported higher Forgotten Joint Scores at 1 year after image‐based robotic‐assisted TKA performed according to functional positioning principles compared with conventional TKA using rKA, while KSS and ROM were similar between groups [22]. This suggests that FKPos may improve subtle aspects of patient‐perceived function, although the clinical relevance of these differences requires further confirmation.

Randomised evidence remains limited. Young et al. reported that FA provided comparable overall outcomes to MA, with a higher proportion of patients willing to recommend the procedure and fewer soft‐tissue releases [42]. These findings support the biomechanical rationale of FKPos but do not yet establish clear clinical superiority.

Overall, available evidence supports the safety and feasibility of FKPos within defined boundaries. Its theoretical advantages are strong, but further prospective comparative studies with longer follow‐up are required to determine its impact on patient satisfaction, function, implant survivorship and revision risk.

## CONCLUSION

FKPos represents an evolution in TKA philosophy, shifting the focus from standardised alignment targets towards integrated, patient‐specific implant positioning. The three‐pillar concept provides a practical framework for this approach by combining bone morphology, ligament behaviour and patellofemoral biomechanics within a single decision‐making process.

Rather than pursuing a universal alignment target, this approach seeks to reconstruct a patient‐specific biomechanical environment that may favour more physiological postoperative knee function.

Image‐based robotic assistance enhances the reproducibility of FKPos by enabling three‐dimensional planning, quantitative gap assessment, controlled component adjustment and dynamic patellofemoral tracking evaluation. Current evidence supports the feasibility and safety of this approach within defined functional safe zones. However, its clinical superiority over other contemporary alignment strategies remains unproven, and long‐term studies are needed to clarify its effect on survivorship and patient‐reported outcomes.

## AUTHOR CONTRIBUTIONS

**Alberto Fogacci**: Literature review; writing—original draft; visualisation. **Clément Favroul**: Literature review; writing—review and editing. **Cécile Batailler**: Conceptualisation; supervision; writing—review and editing. **Elvire Servien**: Supervision; writing—review and editing. **Sébastien Lustig**: Conceptualisation; project administration; supervision; writing—review and editing.

## FUNDING INFORMATION

The authors have no funding to report.

## CONFLICT OF INTEREST STATEMENT

Cécile Batailler: Consultant for Stryker, Smith & Nephew. Elvire Servien: Consultant for Corin. Sébastien Lustig: Consultant for Stryker, Smith & Nephew, Heraeus, Depuy Synthes; Institutional research support from Groupe Lepine, Amplitude; Editorial Board for Journal of Bone and Joint Surgery (Am).

## ETHICS STATEMENT

The authors have nothing to report.

## Data Availability

The authors have nothing to report.
